# Preliminary study of luminescence phenomena from various materials under ultra-high dose rate proton beam irradiation for dose management

**DOI:** 10.1038/s41598-024-65513-3

**Published:** 2024-06-24

**Authors:** Ryosaku Yamada, Teiji Nishio, Daiki Kinkawa, Taketo Tanaka, Mizuki Omura, Yoji Tabata, Hitoshi Yoshimura, Jun Kataoka

**Affiliations:** 1 Medical Physics Laboratory, Division of Health Science, Graduate School of Medicine, Osaka University, Suita-shi, Osaka Japan; 2 Department of Radiology, Kouseikai Takai Hospital, Tenri-shi, Nara Japan; 3 Department of Pure and Applied Physics, Graduate School of Advanced Science and Engineering, Waseda University, Shinjyuku-ku, Tokyo, Japan

**Keywords:** Radiotherapy, Medical imaging

## Abstract

This research aimed to identify materials capable of emitting visible light useful for dose management at ultra-high dose rate (uHDR). Various materials were irradiated with proton beams at a normal dose rate (NDR) and uHDR, and the resulting surface luminescence was captured using a high-sensitivity camera. The luminescence images were compared with the corresponding dose distributions. The luminescence of Tough Water Phantoms (Kyoto Kagaku Co. Ltd.) with various thicknesses was also observed to evaluate the depth distributions. Dose distributions were measured using two-dimensional ionization chamber detector arrays. The Tough Bone Phantom (Kyoto Kagaku Co. Ltd.) exhibited the strongest luminescence among the materials, followed by the Tough Water Phantom. The metals exhibited relatively weak luminescence. The luminescence profiles of the Tough Water Phantom, water, the Tough Lung Phantom (Kyoto Kagaku Co. Ltd.), and an acrylic were similar to the dose profiles. The luminescence distribution of the Tough Water Phantom in the depth direction was similar to that of the dose distributions. The luminescence at uHDR and NDR were approximately equivalent. The Tough Water Phantom was found to be a suitable material for dosimetry, even at uHDR. More detailed measurement data, such as wavelength data, must be collected to elucidate the luminescence mechanism.

## Introduction

Particle therapy using mainly protons and carbon is a type of radiotherapy. Particle therapy is known for its Bragg Peak property, which allows for a more focused dose distribution. Although protons have biological effects similar to those of X-rays, carbon ions are more effective than protons against radioresistant tumors. There are now over 100 particle therapy centers worldwide^1^. As of 2022, over 360,000 people have been treated with particle therapy^2^.

In the field of radiation oncology, there is growing interest in the phenomenon known as the FLASH effect. This effect is triggered by ultra-high dose rate (uHDR) irradiation of over 40 Gy/s with an irradiation time of below 500 ms. It has been reported that compared to normal dose-rate radiotherapy, FLASH has an equivalent anti-tumor effect but provides higher protection to normal tissues^3^. This protective effect is confirmed in treatments at various sites in in-vivo studies^4–7^, and uHDR irradiation on humans has been already conducted^8^.

Accurate dose measurement is essential for radiotherapy under all conditions. Conventionally, radiation detectors include ionization chambers, chemical detectors such as radiochromic films, and luminescence detectors that use light or heat luminescence; each detector has its set of advantages and disadvantages. Luminescence detectors exhibit excellent spatial distribution, time resolution, and dose linearity, even for uHDR. Hence, this type of dosimeter is expected to play a role not only in simple detectors but also in monitoring and three-dimensional dosimetry^9^. Favaudon et al. used this type of dosimeter for the online monitoring of electron FLASH^10^.

Detectors that use visible-light emissions include scintillation and Cherenkov detectors. Scintillation is a phenomenon in which electrons excited by radiation emit light when they return to their ground state. Materials used in detectors include inorganic scintillators such as CsI(Tl), NaI(Tl), and BGO, and organic scintillators, which are made of organic compounds and exhibit high energy transfer efficiency and luminescence. These detectors are already in practical use worldwide.

Cherenkov light is emitted when charged particles pass through a transparent material faster than the speed of light in that medium^11^, and this light emission has a higher time resolution than that of scintillators. This phenomenon is also observed in X-rays and electrons from radiotherapy systems. Cherenkov lights can be used for dosimetric quality assurance and quality control^12–16^, surface-based monitoring system using these lights is implemented^17^. Although their use for particle therapy is also expected, Cherenkov light is not emitted at the distal range of particles because of thresholds. The energies for direct Cherenkov light emissions of protons and electrons are 482 MeV and 0.262 MeV respectively^18^, and the secondary electron above 0.262 MeV requires proton beams above 120 MeV^19^. The proton energies for radiotherapy are approximately 70–250 MeV; therefore, it is believed that the surface luminescence can only be observed^19^.

Recently, several studies have shown that micro-luminescence also occurs when irradiated at energies below the threshold of Cherenkov radiation. Helo et al. reported that Cherenkov light is emitted from electrons originating from prompt gamma X-rays and secondary radioisotopes when water is irradiated with a 60 MeV proton beam^20^. Yamamoto et al. reported that the depth profile luminescence images resembled the simulated dose distributions when irradiated with a 100 MeV proton beam^21^. Yogo et al. reported that luminescence images obtained from irradiated water are practical for uHDR^22^. However, the luminescence of water is very low, and at relatively high energies, Cherenkov light must be subtracted to obtain a luminescence distribution consistent with the dose distribution, which remains difficult to handle.

Because uHDR irradiation transfers the dose instantaneously, the luminescence is also instantaneous. When the head and neck phantom was irradiated with uHDR proton beam, the luminescence phenomenon could be observed with an ordinary camera. (Supplementary Information Fig. S1) Therefore, it may be possible to observe light luminescence that is not detected at a normal dose rate and discover better luminescent material for dose management using uHDR irradiation.This study aimed to discover visible light-emitting materials that can be used in uHDR. Various materials were irradiated with proton beams at a normal dose rate (NDR) and uHDR, and the resulting surface luminescence was captured using a high-sensitivity camera. The luminescence images were compared with the dose distributions.

## Materials and methods

### uHDR proton beam irradiation

**Figure 1:**
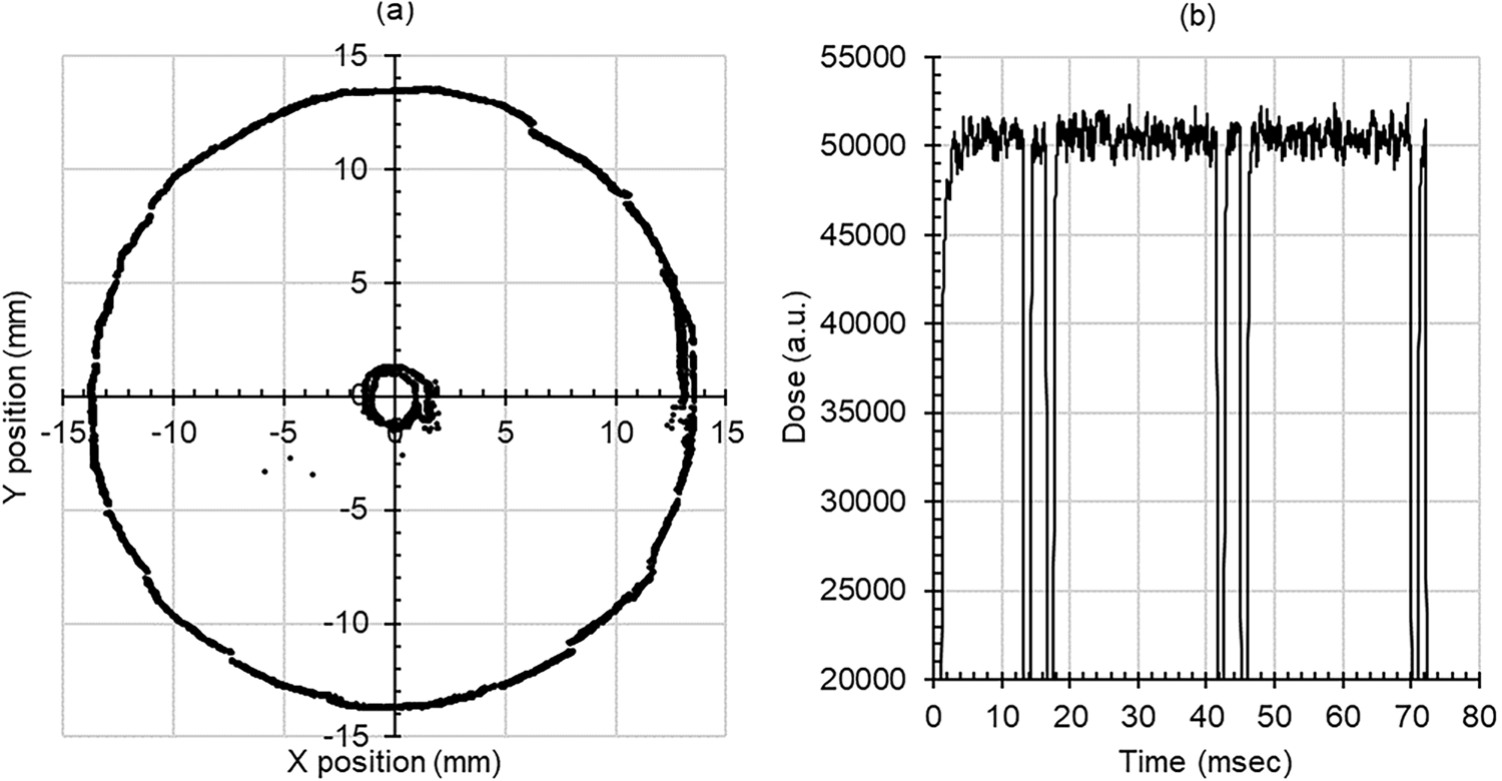
Monitor logs during uHDR irradiation. (**a**) Beam pattern at the profile monitor (**b**) irradiation time at the dose monitor (*uHDR* ultra-high dose rate).

All experiments were performed using proton beams from a proton therapy system with AVF cyclotron (Sumitomo Heavy Industries Ltd., Tokyo, Japan) at the Kouseikai Proton Center (Nara, Japan). The proton beam parameters at uHDR and NDR are listed in Table 1. The beam energy is 230.0 MeV, and the irradiation technique is line scanning. The width of the spread-out Bragg peak (SOBP) was 20.1 mm using a mini-ridge filter. The field size was 23 mmφ at the center of the SOBP. The measured proton beam scan pattern with the profile monitor is shown in Fig. 1a. The two circular scan lines of 27 mmφ and 3.3 mmφ form an irradiation field with a uniform dose distribution at the SOBP center point. The dose rates of uHDR and NDR were 100 Gy/s and 2.06 Gy/s, respectively. The dose rate was adjusted by changing the beam intensity and the number of scans. Figure 1b is the time structure of the uHDR proton beam irradiation as measured by the dose monitor.

**Table 1:** Parameters of the proton beams (*uHDR* ultra-high dose rate, *NDR* normal dose rate).

Proton beams parameter	uHDR	NDR
Energy	230.0 MeV	
Beam scanning	Line scanning (2 ring line scans)	
	(27 mmφ) 7.07 mm/msec (3.3 mmφ) 8.64 mm/msec	
Field size	23.0 mmφ	
SOBP width	20.1 mm	
Dose rate	100 Gy/s	2.06 Gy/s
Irradiation time	72.0 ms	3488 ms

### Luminescence observation system

**Figure 2:**
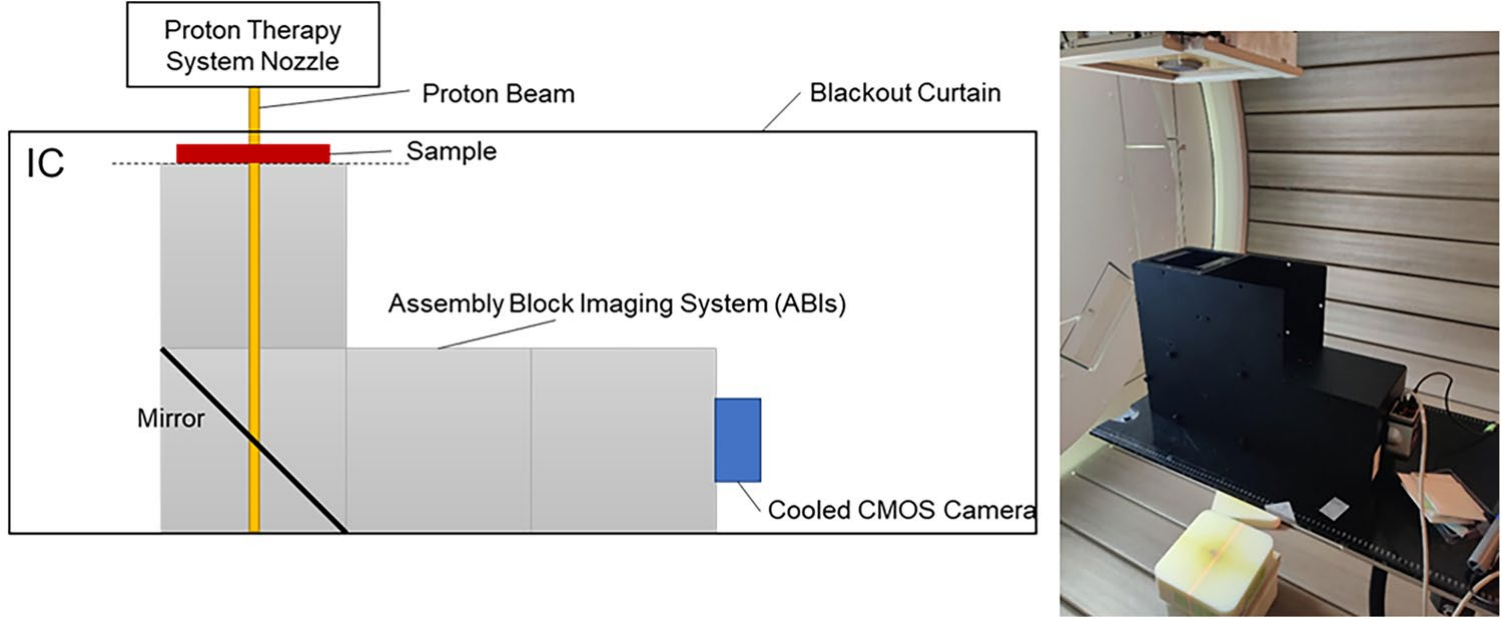
Schematic diagram of the setup of the measurement device and the appearance of the measurement device.

A schematic diagram of the setup of the device used to observe the luminescence and its appearance is shown in Fig. 2. The assembly block imaging system (ABIs) (Osaka University, Osaka, Japan) and irradiated materials were placed below the nozzle of the proton therapy system. ABIs were combined with flames of ABS resin, boards of aluminum, and the mirror and were colored black to minimize self-light emission and unnecessary light reflections, except for the mirror. The interior of the ABIs was dark enough to set each block without any gaps. The samples were placed at the entrance of the ABIs. An isocentric plane was located at the bottom of the sample. Luminescence from the bottom surface of the sample was observed using a cooled CMOS camera (CS-61 M, Bitran Ltd., Saitama, Japan) to reflect the mirror inside the ABIs. The sensitive wavelength band of the camera was 400–1000 nm. The shutter speed of the camera was 30 s, and the gain was 0%. The measurement system was covered with a black curtain to prevent light contamination.

### Measurements of luminescence of various materials

**Figure 3:**
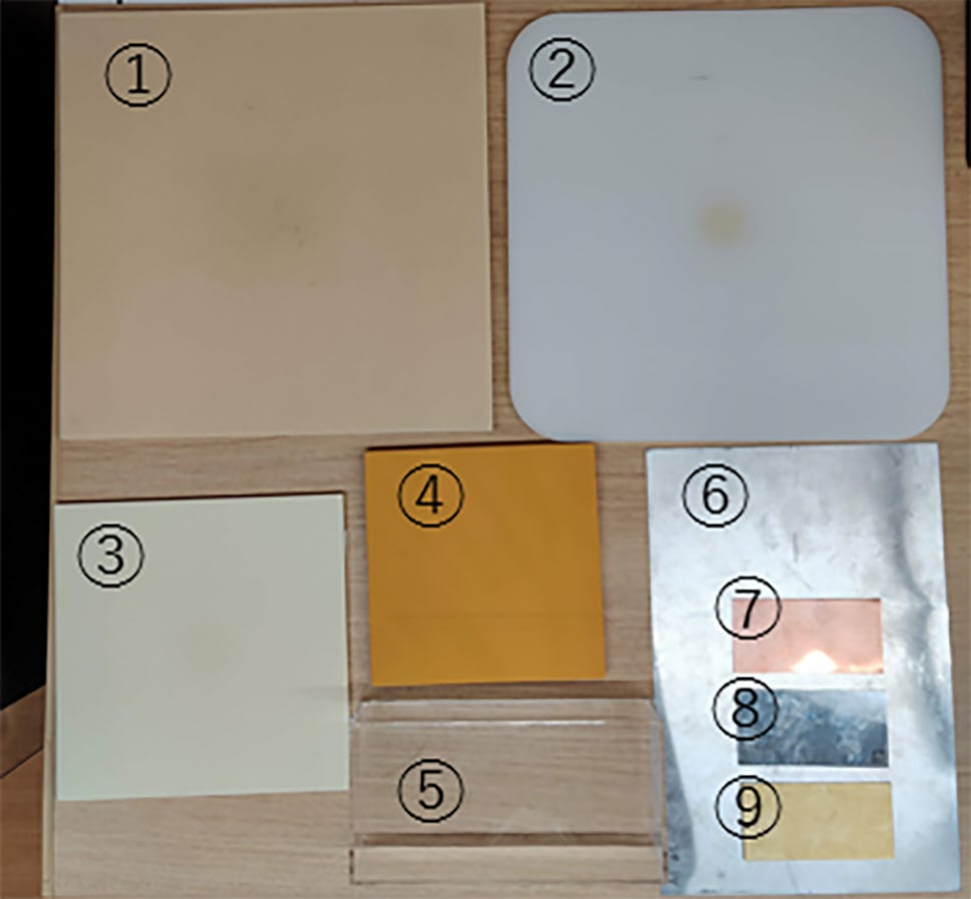
Summary of irradiated materials (Length × Width × Thickness). 1. Tough Water Phantom (300 mm × 300 mm × 10 mm), 2. Polyethylene (300 mm × 300 mm × 10 mm), 3. Tough Bone Phantom (Cortical Bone) (150 mm × 150 mm × 5 mm), 4. Tough Lung Phantom (150 mm × 150 mm × 10 mm), 5. Acrylic case (5 mm thickness), water (depth 10 mm), 6. Aluminum sheet (200 mm × 300 mm × 0.5 mm), 7. Copper sheet (100 mm × 50 mm × 0.5 mm), 8. Brass sheet (100 mm × 50 mm × 0.5 mm), 9. Stainless steel sheet (100 mm × 50 mm × 0.5 mm).

Various materials were irradiated with NDR and uHDR proton beams, and the luminescence of the materials was observed. The irradiated materials are shown in Fig. 3. There were 10 materials: a Tough Water Phantom (WD-3010, Kyoto Kagaku Co., Ltd., Kyoto, Japan), a Tough Lung Phantom (Y04444-97T2-734, Kyoto Kagaku Co., Ltd., Kyoto, Japan), a Tough Bone Phantom (Cortical Bone), (BE-H-05, Kyoto Kagaku Co., Ltd., Kyoto, Japan), polyethylene, an acrylic, water in an acrylic case, an aluminum sheet (A1050), (HA0523, Hikari Co., Ltd., Osaka, Japan), a copper Sheet (C1100P), (CZ553, Hikari Co., Ltd., Osaka, Japan), a brass sheet (YZ552, Hikari Co., Ltd., Osaka, Japan), and a stainless steel sheet (SUS430) (SZ554, Hikari Co., Ltd., Osaka, Japan). Measurements were taken in two patterns. One type of measurement was performed by placing each material alone, and the other type was performed by placing the Tough Water Phantom on top of the material so that the total water equivalent thickness was 310 mm; these measurements were defined as the luminescence at the entrance position and the SOBP position, respectively. The luminescence was observed in the absence of any material to account for the influence of the self-light emission from the ABIs, and the value was used as the background. The irradiation dose was 7.2 Gy at the SOBP position. The values of the region of interest of φ 4 mm to the center of each image were measured as luminescence intensities, and 4 mm wide center profiles were also measured.

### Measurements of dose distributions

**Figure 4:**
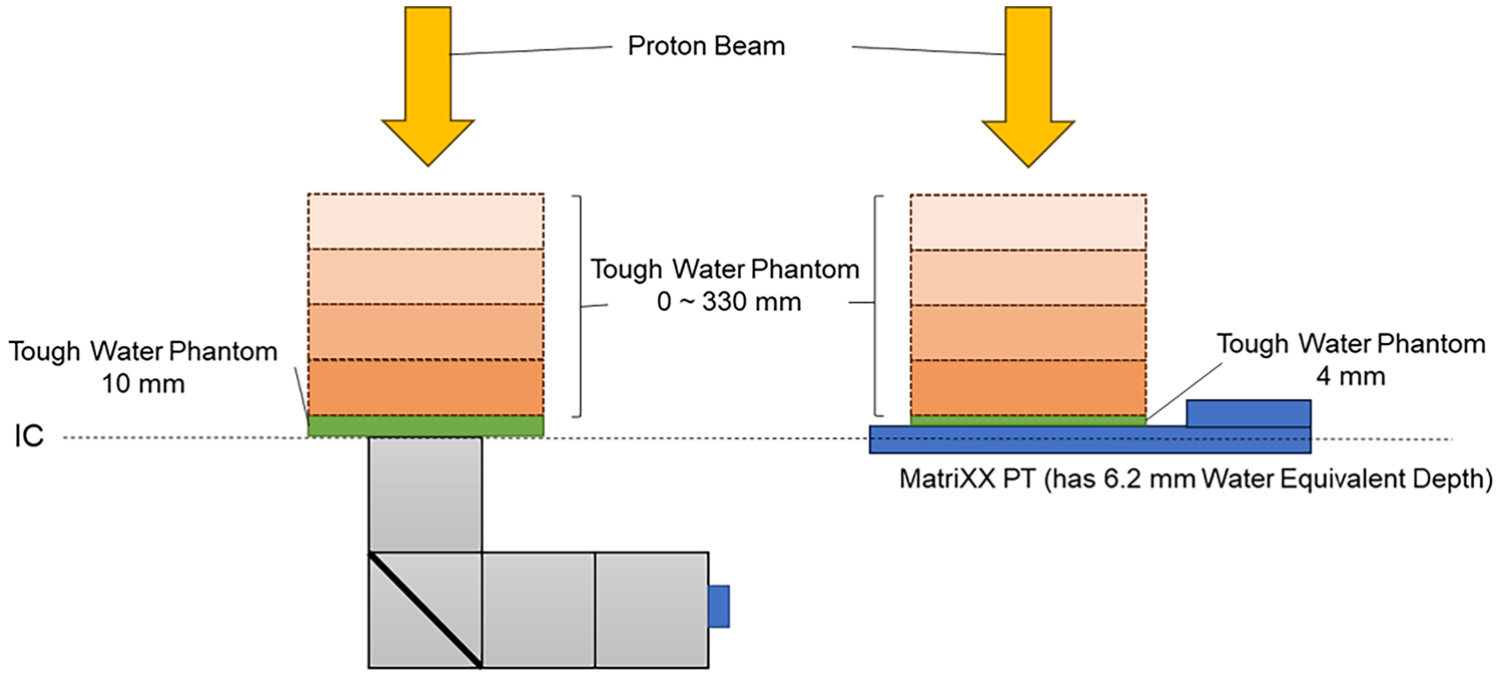
Schematic diagram of measurement of distribution. (Left) Luminescence. (Right) Dose.

Dose distribution was measured with 2-dimensional ionization chamber detector arrays (MatriXX PT; IBA Dosimetry Co., Ltd., Schwarzenbruck, Germany). The use of MatriXX PT at uHDR has not been reported to our knowledge, and we were concerned about failure of this detector. Therefore, MatriXX PT was only used to measure the dose distribution of the NDR proton beam. Furthermore, the agreement between uHDR and NDR doses distribution was confirmed using the parallel plate ionization chamber (Advanced Markus chamber, PTW, Freiburg, Germany). A schematic diagram is shown in Fig. 4 (right). The isocentric plane was the reference point for the chamber, and Tough Water Phantoms with different thicknesses were placed on top of the detector arrays. The detector arrays have a water equivalent depth of 6.2 mm, and the setup in which the Tough Water Phantom of 4 mm was placed was treated as 10 mm. An isocentric plane was the reference point for the detector arrays. The thickness interval was 5 mm in the SOBP region and 10 mm in the other regions. The measurement was also taken with the Tough Water Phantom that was not placed on the arrays. Because of the wide spacing of the chambers of the detector arrays, measurements were also performed by shifting the position of the arrays so that the distance between the measurement points was 3.8 mm. The irradiation dose was 7.2 Gy at the SOBP position.

### Measurements of luminescence of Tough Water Phantom at various thicknesses

The measurements were performed using Tough Water Phantoms of different thicknesses. A schematic diagram of this process is shown in Fig. 4 (left). The Tough Water Phantom at the bottom was always 10 mm thick. Eighteen thicknesses of the Tough Water Phantoms were used: 10, 50, 100, 150, 200, 250, 270, 290, 295, 300, 305, 310, 315, 320, 325, 330, 335, and 340 mm. The irradiation dose was 7.2 Gy at the SOBP position. The values of the region of interest of φ 4 mm to the center of each image were measured as luminescence intensities and 4 mm wide center profiles were also measured.

### Measurements of dose-luminescence relationships of Tough Water Phantom

The measurements were performed using Tough Water Phantoms of two thicknesses, 10 mm and 310 mm, at the entrance and the SOBP positions, respectively. The irradiation doses were 2, 4, 6, 8, 10, 12, 16 and 20 Gy at the SOBP position. Doses at each position were measured using the parallel plate ionization chamber (Advanced Markus chamber, PTW, Freiburg, Germany). Fluence-weighted linear energy transfer (LET) at each position were calculated according to ICRU table^23^.

The values of the region of interest of φ 2.5 mm to the center of each image were measured as luminescence intensities.

### Analysis of measurements

The obtained luminescence images were analyzed using public domain software (Image J, version 1.54d, National Institute of Health, Maryland, USA)^24^. The background image was subtracted from the luminescent image and processed for noise reduction using a median filter. For only the luminescence of water, the luminescent image of the acrylic case was used as the background image. The standard deviation was calculated as the measurement uncertainty.

## Results

**Figure 5:**
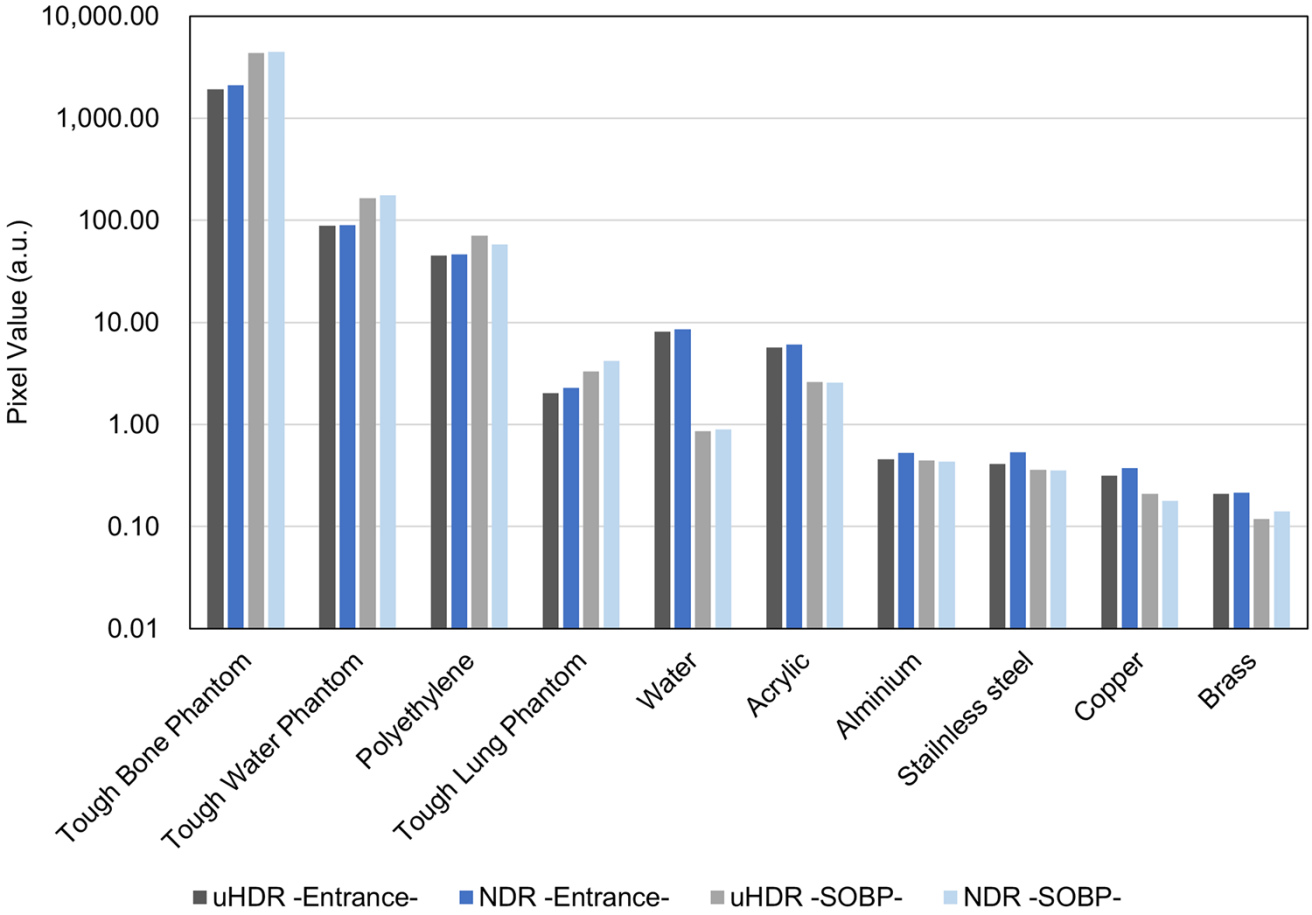
Luminescence intensities of each material at uHDR and NDR. Each value was obtained by setting the region of interest of φ 4 mm in the center of the images (*uHDR* ultra-high dose rate, *NDR* normal dose rate).

Luminescence was observed for all materials. The comparison of luminescence intensities is shown in Fig. 5. The Tough Bone Phantom exhibited the strongest luminescence, followed by the Tough Water Phantom. The metals exhibited weak luminescence, and aluminum and stainless steel exhibited relatively strong luminescence. Non-transparent materials such as the Tough Water Phantom, the Tough Bone Phantom, the Tough Lung Phantom, and polyethylene exhibited stronger luminescence at the SOBP position than at the entrance position. The ratios of luminescence intensities at the SOBP position and the entrance position for each material were 1.92, 1.85, 1.81 and 1.72, respectively. In contrast, the luminescence of transparent materials at the SOBP position was weaker than at the entrance position. The intensities of luminescence at uHDR and NDR were approximately equivalent.

The ratios of doses at uHDR and NDR measured using the parallel plate ionization chamber at the SOBP position, the mid position (220 mm depth), and the entrance position were 1.00, 1.02, and 1.02, respectively. These results led to the conclusion that the dose distributions measured at NDR by the MatriXX PT are consistent with those at uHDR.

**Figure 6:**
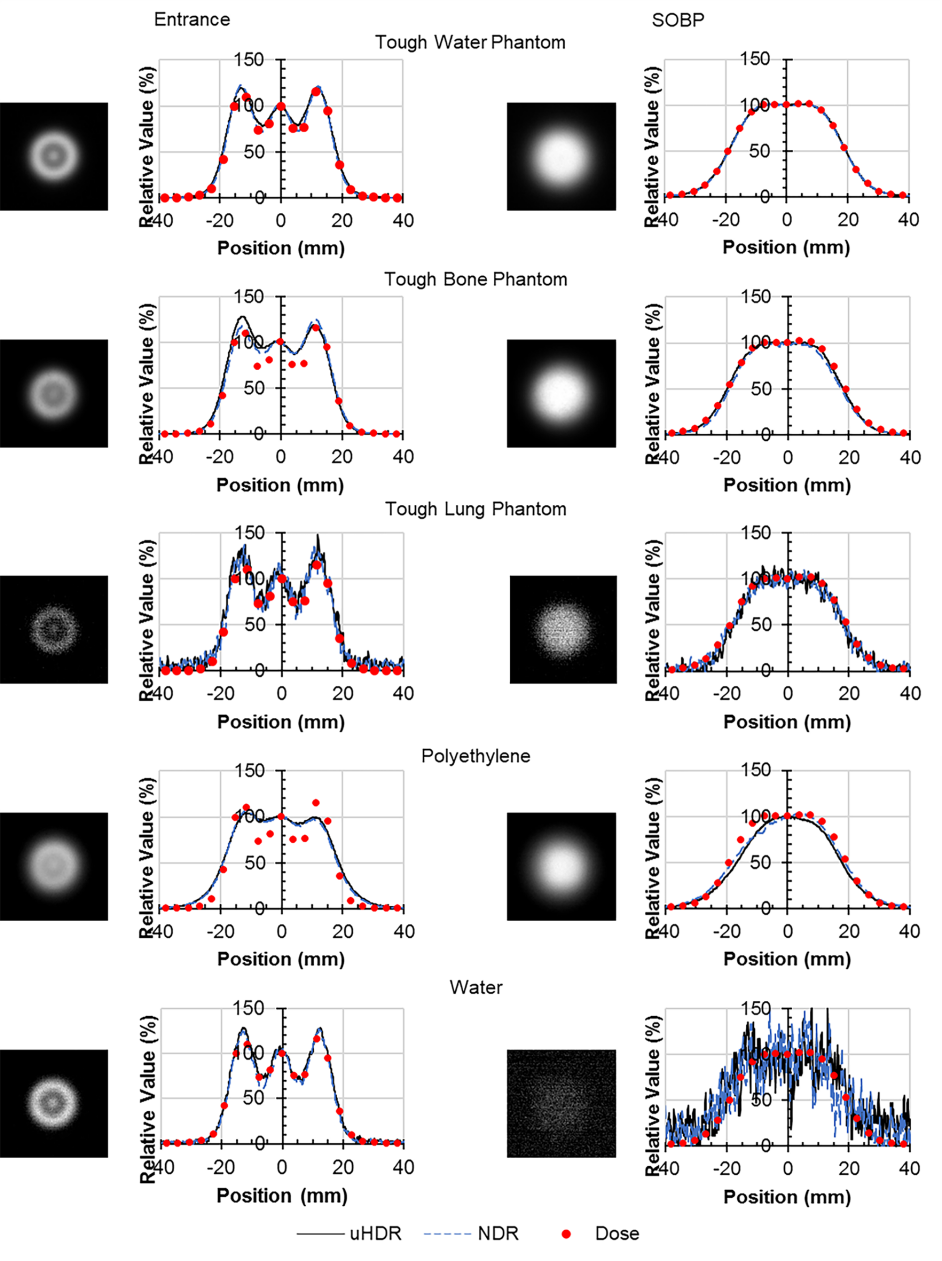

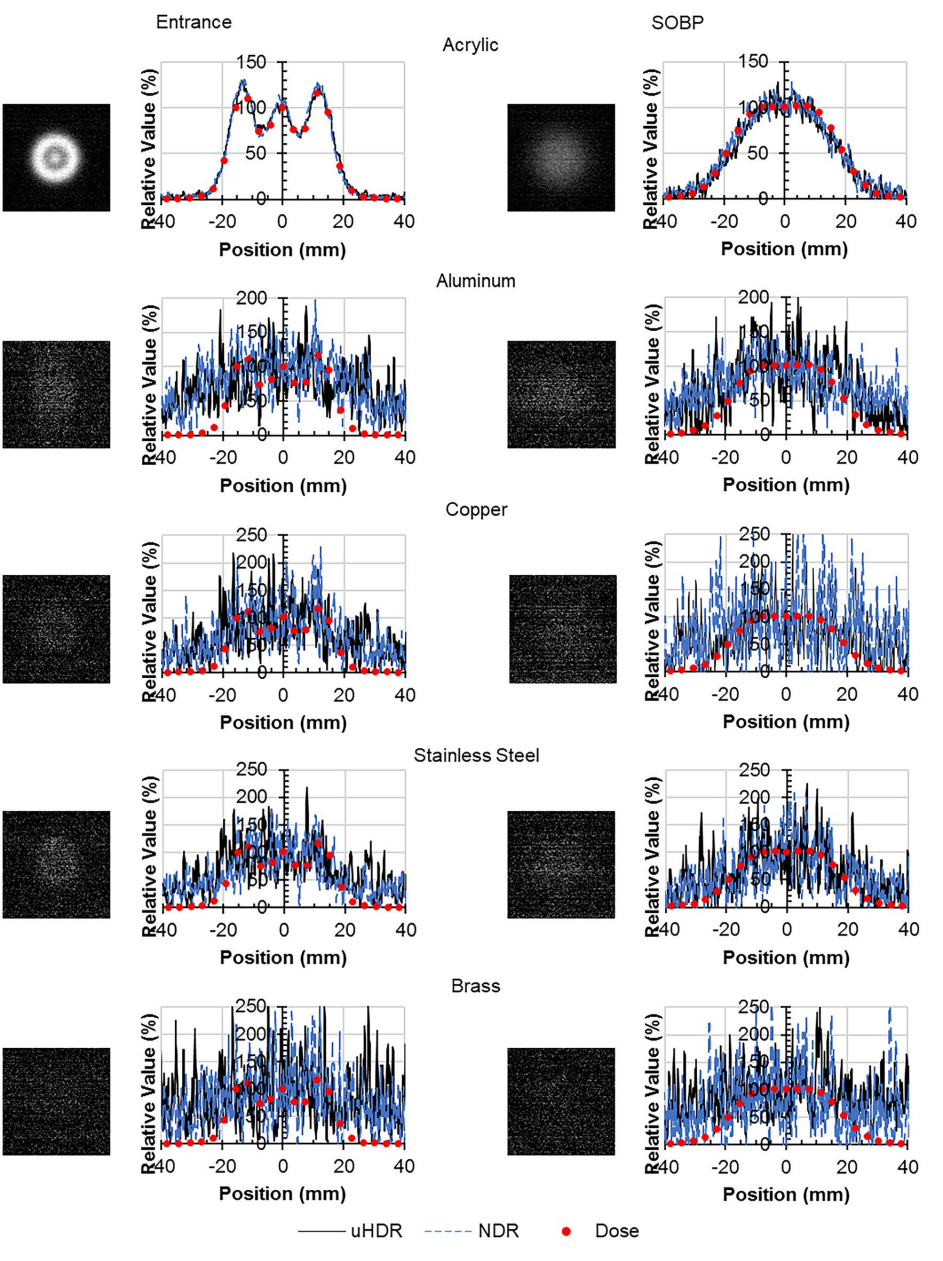
Luminescence images of each material at uHDR and comparison of the luminescence profiles and the dose profiles (left) at the entrance position (right) at the SOBP position (*uHDR* ultra-high dose rate, *NDR* normal dose rate).

The luminescent images at uHDR and profiles at the center of each material are shown in Fig. 6. For comparison, each profile displays the dose profile of the 10 mm Tough Water Phantom at the entrance position and 310 mm Tough Water Phantom at the SOBP position. The profiles were normalized by the value at the 0 mm position.

Although the luminescence profiles of the Tough Water Phantom, water, the Tough Lung Phantom, and the acrylic were similar to the dose profiles, those of the Tough Bone Phantom at the entrance position and polyethylene at both positions were different. Because metals have weak luminescence and a high noise level, a dose comparison was not possible. The luminescence profiles at uHDR and NDR were approximately equivalent.

**Figure 7:**
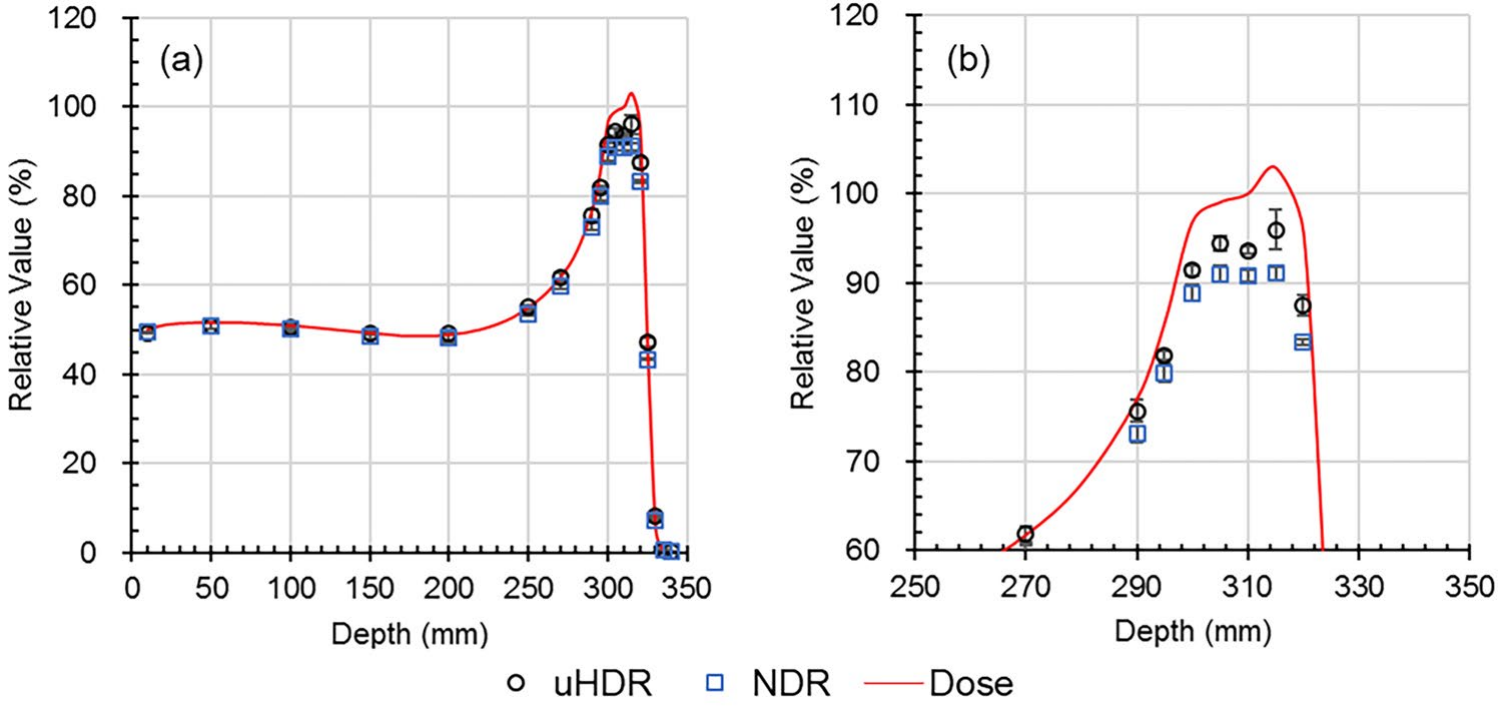
(**a**,**b**) The comparison of depth distributions of luminescence and dose of Tough Water Phantom. The distributions were normalized by the value at 150 mm depth. (**a**) Overall view; (**b**) enlarged view of the SOBP region. Error bars indicate 1 standard deviation (*uHDR*: ultra-high dose rate, *NDR*: normal dose rate). Black circle: uHDR; blue square: NDR; red line: dose.

**Figure 8:**
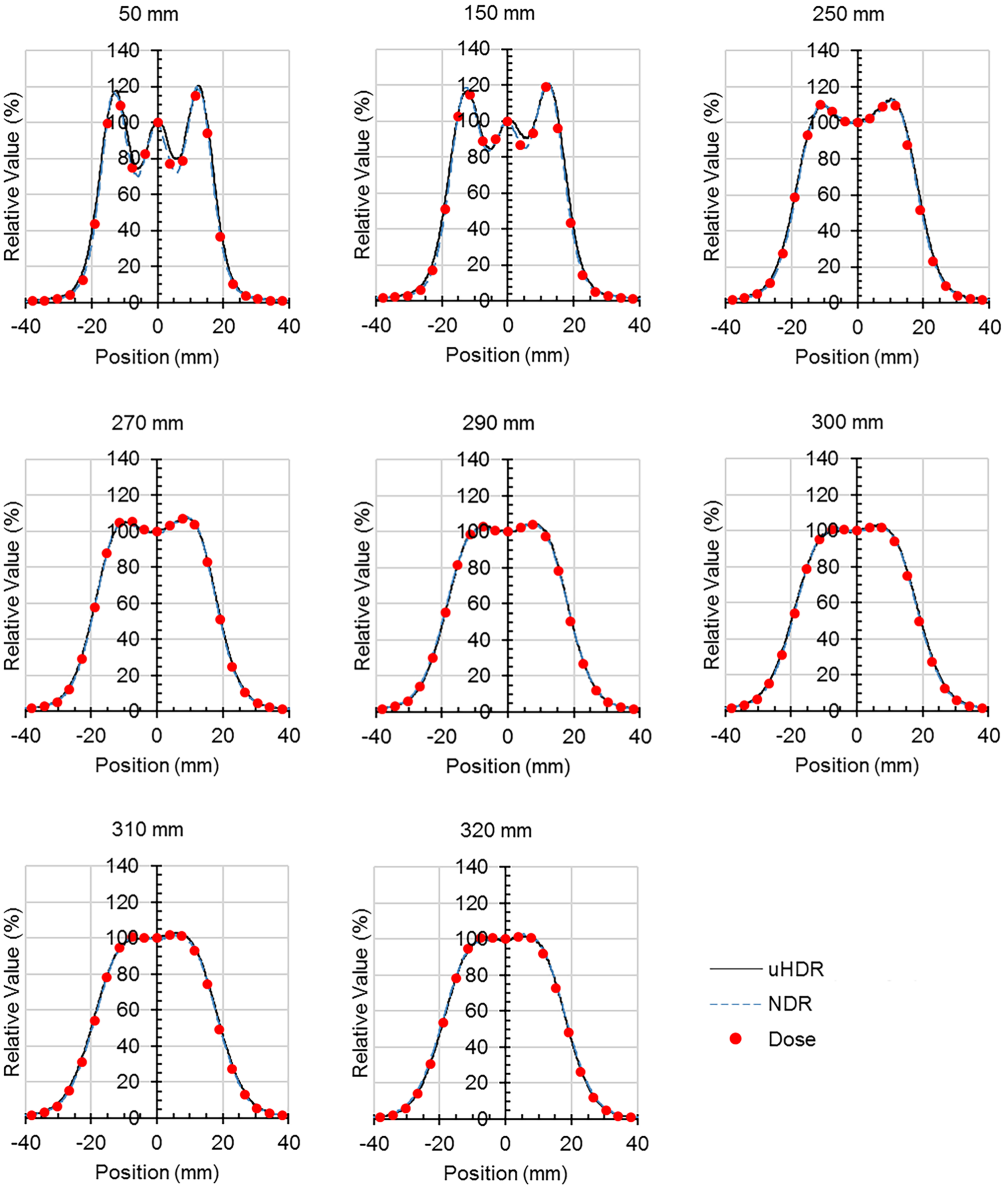
Comparison of profiles of luminescence and dose for various thicknesses of Tough Water Phantoms. The profiles were normalized by the value at the center position (*uHDR* ultra-high dose rate, *NDR* normal dose rate). Black line: uHDR blue dashed line: NDR red dot: dose.

Comparisons of the luminescence profiles of the Tough Water Phantom with various thicknesses and dose profiles are shown in Figs. 7 and 8, respectively. Lateral profiles are shown in excerpts. The depth distributions were normalized by the value at a depth of 150 mm, and the profiles were determined by the value at the center. The depth distribution of luminescence was the list of the values of the region of interest of φ 4 mm to the center of each image. Although the depth distribution of the luminescence was generally comparable to the depth dose distribution, there was a difference in the SOBP position. The luminescence profiles were similar to the dose profiles for all thicknesses. The depth distributions and luminescence profiles for uHDR and NDR were comparable.

**Figure 9:**
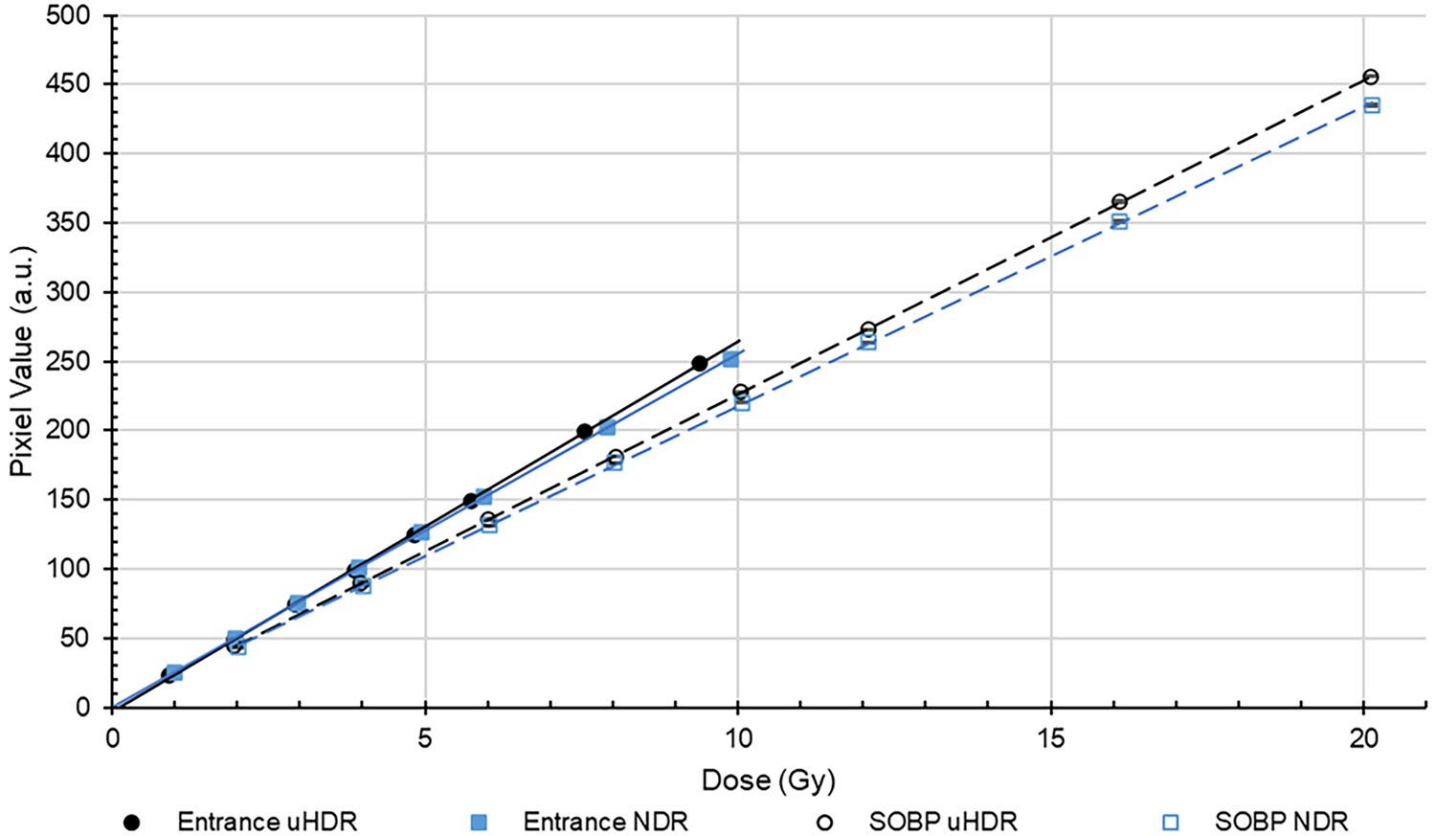
Relationships between luminescence and dose of the Tough Water Phantom. Error bar indicates 1 standard deviation (*uHDR* ultra-high dose rate, *NDR* normal dose rate). Black circle: uHDR; blue square: NDR; straight line: the entrance position; dashed line: the SOBP position.

The luminescence intensities of the Tough Water Phantom at various doses are shown in Fig. 9. A linear relationship between luminescence and dose was observed in all conditions. The coefficients of determination were all greater than 0.99. The slopes of the approximate lines at uHDR entrance, NDR entrance, uHDR SOBP, and NDR SOBP were 26.7, 25.5, 22.7, and 21.9, respectively. The intensities at the SOBP position were weaker than at the entrance position. The slopes of approximate lines at the SOBP position at uHDR and NDR were 15.1% and 15.2% smaller than at the entrance position, respectively. The intensities at both positions for uHDR were slightly stronger than for NDR at relatively high doses. The slopes of the approximate lines at uHDR at the SOBP position and the entrance positon were 4.5% and 4.4% stronger than at NDR, respectively. Fluence-weighted LET at the entrance and the SOBP position were 4.2 and 17.8 MeV g^–1^ cm^2^, respectively. Dose measurement uncertainties were all less than 0.5% at one standard deviation.

## Discussion

Visible light luminescence was observed for all irradiated materials. We considered which material was the most suitable for dosimetry. The dose at the SOBP position was about twice that at the entrance position, and the ratios of the Tough Water Phantom and the Tough Bone Phantom were in good agreement with the dose. This is thought to be because the main component of luminescence is scintillation. Luminescence intensities at water and the acrylic at the SOBP positions were smaller than at the entrance. This is probably because most of these luminescence components are Cherenkov light with an energy threshold. It has been reported that the luminescence of acrylic irradiated with 2.7 MeV electron beams is mostly due to Cherenkov radiation^25^. Luminescence distributions of the Tough Water Phantom, the Tough Lung Phantom, water, and the acrylic were comparable to the dose distributions. The Tough Bone Phantom and polyethylene also exhibited strong luminescence; however, distributions differed from the dose distributions. The Tough Bone Phantom included P and Si, whereas the Tough Water Phantom did not. Polyethylene may also contain additives. Simplified measurements using optical filters were performed for these three materials, and it was also confirmed that the luminescence wavelengths were different for each. (Supplementary Information Fig. S2) These differences are thought to be due to differences in the composition of these substances. These results suggest that the Tough Water Phantom is the most suitable material for dose management among materials irradiated in this study.

More detailed experiments were conducted on the Tough Water Phantom. The depth distributions of the Tough Water Phantom were similar to those of the doses and linearity between luminescence and dose was observed. However, one difference between the two was that the luminescence intensities at the SOBP position were slightly weaker than at the entrance position. There was also a difference in the calculated LET values for the two positions. This can be attributed to the quenching of luminescence; in other words, the LET dependence. The intensities at both positions for uHDR were slightly stronger than for NDR at relatively high doses. This is related to the CMOS camera’s threshold, and is thought to be because uHDR produces strong instantaneous luminescence. The CMOS camera sensor will not output a light-receiving signal unless the input light intensity per hour is above a certain value. Because uHDR emits stronger light instantaneously, the percentage of total light emission that exceeds the camera threshold is considered to be higher than that of NDR. It could also be due to the uncertainty of the ionization chamber.

These results indicate that the Tough Water Phantom has strong visible light luminescence, which is similar to the dose distribution of proton beams with high dose rates. The luminescence of the Tough Water Phantom can be used to provide accurate dosimetry for proton irradiations at different dose rates, although the LET dependence should be noted.

Several concerns can be considered. Plastic materials such as those in the Tough Water Phantom are damaged by repeated irradiation. This may change the dose-luminescence scale or luminescence distributions. In addition, radioactivation also occurs. The Tough Water Phantom is composed of a greater number of elements than polyethylene and water, so more attention should be paid to this problem. The luminescence observation system used in this study emitted visible light without any material placed on it. This was considered to be caused by luminescence originating from the mirror in this system. This luminescence is considered to include air luminescence. However, air luminescence is much weaker than the luminescence of water^26^ and therefore almost impossible to measure with the system used in this study. Although this effect is suppressed by the image subtraction process, this was considered as one of the factors of uncertainty in this study.

In FLASH proton therapy with uHDR proton irradiation, information of the light emission distribution can be used to monitor the proton irradiation area on the patient’s body surface. In the future, we plan to perform experiments for imaging of the luminescence distribution on animal skin by uHDR proton irradiation.

## Conclusion

We observed the visible light luminescence of various materials when irradiated with proton beams at NDR and uHDR with a high-sensitivity camera and compared their luminescence images with the dose distributions. Visible-light luminescence was observed in all irradiated materials, and the Tough Water Phantom was found to be a suitable material for dosimetry despite its LET dependence. The small dose-rate dependence of the proton excitation fluorescence properties of Tough Water indicated that it potentially is as useful for dosimetry in the uHDR region as it is in the NDR region. Water-equivalent phantoms such as the Tough Water Phantom exist in almost all radiotherapy facilities, and this dose management system can be established relatively easily in existing facilities. More detailed measurement data must be collected to elucidate the luminescence mechanism.

### Supplementary Information

Supplementary Figures.

## Data Availability

The datasets used and/or analysed during the current study available from the corresponding author on reasonable request.
