# Supplementary material for: Preliminary study of luminescence phenomena from various materials under ultra-high dose rate proton beam irradiation for dose management
Source: Sci Rep. 2024 Jun 24;14:14504. doi: 10.1038/s41598-024-65513-3 (PMC11196681; doi:10.1038/s41598-024-65513-3)
Supplement: Supplementary file 1 — Supplementary Figures. [file 41598_2024_65513_MOESM1_ESM.docx]

Supplementary Information

**Preliminary study of luminescence phenomena from various materials under ultra-high dose rate proton beam irradiation for dose management**

**Ryosaku Yamada1,2, Teiji Nishio1,*, Daiki Kinkawa1, Taketo Tanaka1, Mizuki Omura1, Yoji Tabata2, Hitoshi Yoshimura2, Jun Kataoka3**

^1^ Medical Physics Laboratory, Division of Health Science, Graduate School of Medicine, Osaka University, Suita-shi, Osaka, Japan.

^2^ Department of Radiology, Kouseikai Takai Hospital, Tenri-shi, Nara, Japan.

^3^ Department of Pure and Applied Physics, Graduate School of Advanced Science and Engineering, Waseda University, Shinjyuku-ku, Tokyo, Japan.

*nishio@sahs.med.osaka-u.ac.jp


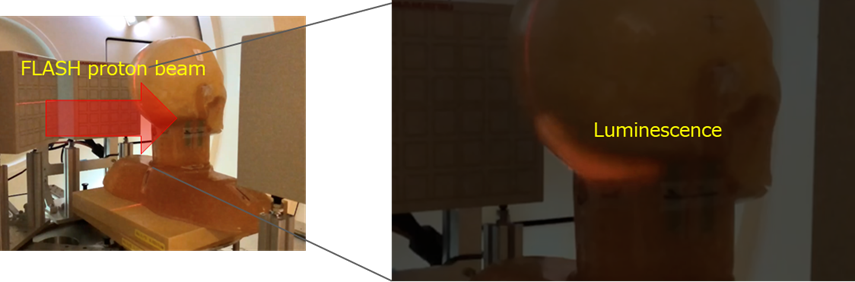


**Figure S1.** Results of pre-experiment of ultra-high dose rate (uHDR) proton irradiation to a urethane-based head and neck phantom at Nagoya Proton Therapy Center (Aichi, Japan).


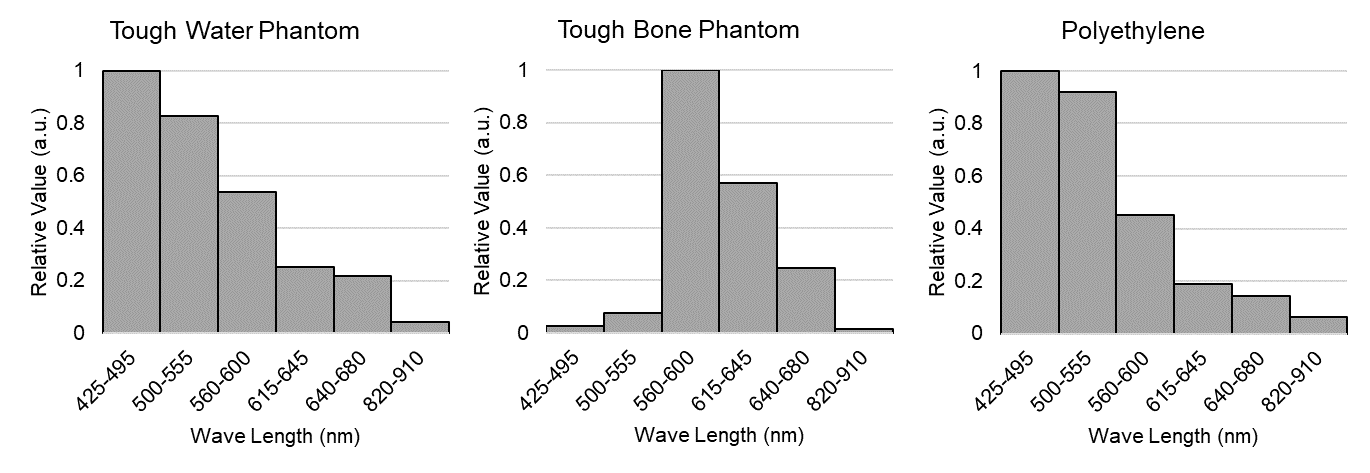


**Figure S2.** Results of luminescence measurements using optical filters for each wavelength in uHDR proton irradiation to a Tough Water phantom, Tough Bone phantom, and polyethylene.
